# How Healthcare Systems Negatively Impact Environmental Health? The Need for Institutional Commitment to Reduce the Ecological Footprint of Medical Services

**DOI:** 10.3390/epidemiologia4040043

**Published:** 2023-11-22

**Authors:** Prisco Piscitelli, Stela Karaj, Alessandro Miani, Tassos C. Kyriakides, Enrico Greco, Elena Colicino, Antonio Bray, Fernando Simón, Vasilis Vasiliou, Andrea A. Baccarelli

**Affiliations:** 1 Italian Society of Environmental Medicine (SIMA), Viale di Porta Vercellina, 9, 20123 Milan, Italy; prisco.piscitelli@unisalento.it (P.P.); enrico.greco@units.it (E.G.); 2 Department of Experimental Medicine, University of Salento, 73100 Lecce, Italy; 3 Local Health Authority ASL Le, 73100 Lecce, Italy; dirsan@asl.lecce.it; 4 Faculty of Social Sciences, European University of Tirana, 1000 Tirana, Albania; 5 Department of Biostatistics, Yale School of Public Health, New Haven, CT 06510, USA; tassos.kyriakides@yale.edu; 6 Department of Chemical and Pharmaceutical Sciences, University of Trieste, 34127 Trieste, Italy; 7 Department of Environmental Medicine and Public Health, Ichan School of Medicine, Mount Sinai Hospital, New York, NY 10029, USA; elena.colicino@mssm.edu; 8 Spanish Ministry of Health, 20014 Madrid, Spain; fsimon@sanidad.gob.es; 9 Department of Environmental Health, Yale School of Public Health, New Haven, CT 06510, USA; vasilis.vasiliou@yale.edu; 10 Harvard T. H. Chan School of Public Health, Boston, MA 02115, USA; abaccarelli@hsph.harvard.edu

The global healthcare industry plays a crucial role in preserving human health and well-being. However, there is a growing concern that the operation of healthcare systems may have unintended negative consequences on environment and health. Actually, healthcare systems worldwide are aimed at improving human health and prolonging life expectancy, but the pursuit of better health outcomes has environmental ramifications that are often underperceived [1,2,3,4,5,6,7].

In Western countries, the health sector represents between 8 and 10% of a country’s gross domestic product and employs 8% of total workers. This large-scale activity inevitably results in having a huge impact on the environment since it requires the use of various means of transportation, and the consumption of electricity and chemicals. Therefore, it is not a surprise that healthcare systems account for an average of 8.5% of total greenhouse gas emissions in the United States and about 6% in other Western countries [1]. Specifically, in a 2013 study, the US healthcare sector was found to be responsible for 12% of the overall national acid rain emissions, 10% of greenhouse gas emissions recorded that year and 10% of smog formation, being responsible also for 9% of air pollutants (including carcinogenic toxics) and 1% of stratospheric ozone depletion.

Europe, USA and China account for over half of the world’s healthcare-related emissions [1,6]. The British Health Service alone emits 25 million tonnes of CO_2_ annually [2]. These emissions of UK are equivalent to the annual emissions of the entire Croatia and represent a quantity of CO_2_ similar to that emitted by 12 million vehicles travelling an average distance of 15,000 km in a year [2]. Additionally, the healthcare sector consumes 39 billion litres of water every year, which is twice the capacity of the renowned Lake of Como (in Italy) and more than half of Geneva or Lausanne’s lakes (in Switzerland).

The structure of a hospital is complex as it consists of people, institutions and resources that produce an environmental impact at all levels on air, water and soil (as well as in terms of radiation risk). The main sources of emissions are the buildings and machinery used for the care of patients. 

According to the European RES-Hospitals project, which aims to reduce the carbon dioxide emissions of hospitals among EU Member States, the CO_2_ production of hospital buildings across Europe represents 5% of total greenhouse emissions.

Hospitals need an uninterrupted supply of energy for heating and cooling, ventilation, machinery, healthcare treatments and cleaning. Additionally, every hospital must have an alternate power generator for blackout situations. At a global level, the greenhouse emissions of healthcare systems are equivalent to those of 514 coal-fired power plants.

Another huge impact is that generated by drugs. A study performed by the “BIO Intelligence Service” of the European Environmental Agency, carried out for the European Union in 2013 in the frame of a “One Health” perspective, has computed that from 30 to 90% of the oral dose of any drug is released into the environment through urine. From water, these residues can spread onto the surface and onto cultivated lands, until they reach running water and the foods we eat. In fact, hospital waters, the main sources of micro-pollutants, are generally not separated from urban ones, and the purification plants used to treat them are unsuitable for removing the specific components present in the discharges of healthcare facilities.

In addition to the residues disseminated in wastewater, another huge impact is that produced by wastes: for the year 2017, the Italian healthcare system alone produced about 792,827 tons of wastes (6% of the total national waste production), which were classified as hazardous in 75% of cases. About 77% of these wastes come from the pharmaceutical industry. This happens despite the capability of the same industries of adopting specific measures which are effective in reducing this kind of environmental impact, mainly consisting of the production of biodegradable wrappings and different sizes of medicine boxes (depending on the type of treatment needed). Doctors, on the other hand, should reduce the prescription of antibiotics (which represents a real emergency in veterinary medicine). Moreover, expired or unused medicines should be correctly managed for proper disposal. The same goes for the use of diagnostic kits and laboratory tests that could facilitate significant water savings. Clinical chemistry and immunodiagnostic laboratory systems alone require an average water supply of 26 L/h, thus resulting in up to 200,000 L/year, despite the availability of systems that do not require a water supply because they do not use fixed probes for sample or reagent dispensing and do not involve any decontamination procedures.

In addition, much more can be accomplished in the field of energy self-production at the hospital level. A study by the Commonwealth Fund estimated that, in the US, small energy management interventions—such as the control of lighting through automation systems or the use of ecological cleaning products—can generate savings of USD 15 billion over a 10-year period. In 2011, the German NGO BUND launched the “Energy Savings Hospitals” program, which consists of a recognition for those hospitals that are committed to reducing the energy consumed. So far, a total of 45 hospitals have received this award as they have avoided the emission of 65 thousand tons of CO_2_ per year, generating savings of more than 20 million euros. In France, the Centre Hospitalier de Niort has focused on solar energy since year 2014, such that this hospital is now able to produce its own energy and generate a surplus of electricity that can be used for other purposes. This experience shows how photovoltaic panels and the maximization of the use of natural light can contribute to increase the sustainability of healthcare facilities.

In Bologna, (Italy), the Emilia-Romagna Regional Government launched the “Regional Health Service for Sustainable Development” program about 10 years ago, aimed at reducing the environmental burden of the regional healthcare service and to promote rational use of energy. This pilot points out that interventions impacting daily hospital activities should not be underestimated: over the course of a week, turning off the air conditioning just half an hour before leaving the office is equivalent to 24 h of electricity consumed by one television; a photocopier turned on outside working hours for a week consumes as much as it does printing 8500 photocopies; over the course of a year, using the toilet flush with the double button is equivalent to the water of 33 showers. 

These actions assume great importance if multiplied by the 61,000 employees and the approximately four million square meters of the public health facilities present in the Emilia-Romagna region alone. Specifically, a mere 1% drop in energy consumption can lead to savings of EUR one million per year and reduce CO_2_ emissions by about 4000 tons annually. Another aspect which the healthcare system must care about is the reduction in food wastes. In the same city of Bologna, at St. Orsola University Hospital, open foods not consumed by patients are currently used to produce biogas and generate electricity.

In the United States, about 1200 hospitals are members of Practice Greenhealth, an organization supporting sustainability solutions, but they represent only 20% of US hospital facilities, despite the sustainability measures adopted in Minnesota and Wisconsin by eight different hospitals since 2013 have shown to generate average savings of USD 2.5 million annually. Success stories include safer medication disposal, reduced paper use, the embracing of telemedicine and the installation of solar panels. 

However, these experiences cannot remain at the level of “pilot projects”, and the reduction in the environmental footprint of healthcare systems (starting from hospitals) should soon be fostered through the adoption of specific legislation at local, national and international levels. Health-care facilities in USA are employing many strategies to cut waste and lower emissions. This commitment includes a variety of actions ranging from water conservation and waste management efforts, to switching to electronic medical records (EMRs), and purchasing and using non-toxic and reusable cleaning supplies, as well as becoming increasingly aware of medical services’ environmental impact. On October 24, The International Hospital Federation (IHF) of Geneva launched its Sustainability Accelerator Tool to help hospital and healthcare leaders assess and track organizational efforts to reduce emissions and promote environmental stewardship. As a founding member of the IHF, the American Hospital Association has agreed to help promote this tool among US hospitals. Also, the National Academy of Medicine has set a Climate Collaborative Delivery Working Group, which developed a shortlist of key actions for US hospitals and health systems to reduce their greenhouse gas emissions. 

The United Nations (UN) plays a pivotal role in the global healthcare framework by actively participating in diverse agencies, initiatives and programs. Its engagement with healthcare systems worldwide primarily focuses on promoting well-being, tackling health inequalities and enhancing the availability of healthcare services. This commitment extends to “addressing global challenges related to economics, social well-being, and healthcare”. Formally, the United Nations (UN) have included the environmental sustainability of healthcare systems among the seven priority fields upon which we have to focus in order to protect our health and the environment [8].

The new challenge is to “bring the culture of the environment into healthcare s ystems” [9]. A change in perspective is necessary to fulfil this goal. In fact, enhancing patient knowledge in the frame of primary prevention, shaping attitudes, and equipping individuals with specific skills might play a pivotal role in supporting patients in managing their health concerns, ultimately leading to improvements in well-being, satisfaction and the recovery process. In addition, health education can potentially contribute to a reduction in healthcare costs and environmental impact. In recent years, there has been a growing emphasis on preventive and educational dimensions of healthcare, with family medicine serving as a cornerstone in fostering health-oriented mindsets, in order to reduce hospitalizations and its global burden [10].

Indeed, according to the approach promulgated by the UN 2030 Agenda, health is closely related to the social, economic, and cultural context in which it is included and, consequently, the sustainability of healthcare systems cannot ignore these factors. This Agenda represents a strategy encompassing individuals, the environment and economic well-being, calling for such challenging objectives as the eradication of AIDS, tuberculosis, malaria and other communicable diseases by 2030, as well as the extension of universal health coverage to ensure every person has access to safe and reasonably priced medicine.

However, this will naturally boost investment in healthcare systems, which could translate into further damage to the environment and consequently to human health if the issue of sustainability is not considered. Unfortunately, only a half of our healthcare organizations have included the UN Sustainable Development Goals (SDGs) in their strategic plan, and only half of these organizations declared that they had included the Sustainable Development Goals in their strategic plan.

The indicators to monitor the country’s positioning with respect to Objective number 3 of the 2030 Agenda still need to be developed both at the national level and healthcare organization level to measure their sustainability performance. The transition towards the sustainability of healthcare systems will therefore depend on the ability to seize these opportunities, through the implementation of substantial interventions that are able to guarantee the achievement of the targets of the UN 2030 Agenda for Sustainable Development. We all have to keep in mind that “taking care of people and taking care of the planet are two sides of the same coin”, and that places of healing and care should not themselves constitute a risk factor for health.
